# Application of Macro-Instrumented Indentation Test for Superficial Residual Stress and Mechanical Properties Measurement for HY Steel Welded T-Joints

**DOI:** 10.3390/ma14082061

**Published:** 2021-04-19

**Authors:** Junsang Lee, Kyungyul Lee, Seungha Lee, Oh Min Kwon, Won-Ki Kang, Jong-Il Lim, Hee-Keun Lee, Seong-Min Kim, Dongil Kwon

**Affiliations:** 1 Department of Materials Science and Engineering, Seoul National University, Seoul 08826, Korea; tpflwkdhk@snu.ac.kr (J.L.); lky0112@snu.ac.kr (K.L.); lshaa@snu.ac.kr (S.L.); kwonohmin7@snu.ac.kr (O.M.K.); 2 Naval & Special Ship Structural Design Department, Daewoo Shipbuilding & Marine Engineering CO., LTD., Geoje 53302, Korea; sojoong33@dsme.co.kr (W.-K.K.); jilim@dsme.co.kr (J.-I.L.); 3 Welding Engineering R&D Department, Daewoo Shipbuilding & Marine Engineering CO., LTD., Geoje 53302, Korea; zetlee@dsme.co.kr

**Keywords:** high-yield-strength steel, welding, instrumented indentation test, yield strength, residual stress

## Abstract

HY-80 and HY-100 steels, widely used in constructing large ocean vessels and submarine hulls, contain mixed microstructures of tempered bainite and martensite and provide high tensile strength and toughness. Weld integrity in HY steels has been studied to verify and optimize welding conditions. In this study, the T-joint weld coupons, HY80 and HY100, were fabricated from HY-80 and HY-100 steel plates with a thickness of 30 mm as base metals by submerged-arc welding. Flux-cored arc welding was performed on an additional welding coupon consisting of HY-100 to evaluate the effect of repair welds (HY100RP). Microstructures in the heat-affected zones (HAZ) were thoroughly analyzed by optical observation. Instrumented indentation testing, taking advantage of local characterization, was applied to assess the yield strength and the residual stress of the HAZ and base regions. The maximum hardness over 400 HV was found in the HAZ due to the high volume fraction of untempered martensite microstructure. The yield strength of the weld coupons was evaluated by indentation testing, and the results showed good agreement with the uniaxial tensile test (within 10% range). The three coupons showed similar indentation residual stress profiles on the top and bottom surfaces. The stress distribution of the HY100 coupon was comparable to the results from X-ray diffraction. HY100RP demonstrated increased tensile residual stress compared to the as-welded coupon due to the effect of the repair weld (323 and 103 MPa on the top and bottom surfaces). This study verifies the wide applicability of indentation testing in evaluating yield strength and residual stress.

## 1. Introduction

HY-80 and HY-100 steels are quenched and tempered low-alloy steels that were developed for the construction of large ocean vessels and submarine hulls [1]. HY-100 has a higher range of nickel content (2.25–3.50%) than HY-80 (2.00–3.25%), with the same proportions of other elements [1]. As a result of quench hardening followed by tempering, HY series steels show mixed microstructures of tempered bainite and martensite [2,3]. These steels are designed to provide better tensile strength and toughness with high ductility compared to conventional carbon steels with lower weight, and they offer better structural performance. They provide superior strength per weight than conventional structural steels and yield cost savings by simplifying or eliminating heat treatment processes [3,4,5,6]. Hydrogen embrittlement was investigated due to the offshore application of these steels [7,8,9]. The effect of welding on the mechanical properties of these steels has been studied with conventional uniaxial tensile testing, the Charpy V-notch impact test, and a hardness test [3,6,10,11], and optimum welding parameters for gas metal arc welding have been proposed [11]. The welding residual stress has been evaluated and analyzed with experimental stress measurement techniques and finite element methods [12,13,14,15]. Repair welding is commonly used to rejoin material when cracks are observed adjacent to weld joints. Many studies have shown the effect of repair welding on residual stress distribution in different materials [16,17,18,19]. The effects of the stress and welding on the J-R curve and crack propagation have also been investigated [20,21,22,23,24].

The instrumented indentation technique has been proposed to measure the surface mechanical properties of small-scale materials such as thin films and coated components [25]. Methods for evaluating various mechanical properties, including hardness, elastic modulus, tensile properties, and fracture toughness [25,26,27,28,29], have been proposed. Over the past 20 years, much research has been conducted to apply indentation testing to large-scale components by taking advantage of the semi-destructive and local nature of indentation. In particular, tensile properties and the residual stress around the welding heat-affected zone have been investigated [30,31,32,33,34,35].

Much work has been done on evaluating tensile properties by indentation testing. After Tabor [36] empirically related the uniaxial tensile stress–strain to indentation parameters using a spherical indenter, many studies refined the form of the relationship. Modification of strain definition and the analysis of pile-up effects [27], functionalization of a plastic constraint factor by the strain-hardening exponent [37,38], and applications and optimizations for specific materials [39,40] were studied. A method to match stress–strain data from indentation testing with uniaxial tensile testing originated from the study of the stress–strain field beneath the indenter [41]. This study analyzed the elastoplastic deformation assuming a hemispherical core expanding in the material and also hydrostatic pressure and perfect core plasticity without considering pile-up (expanding cavity model). This model was extended by introducing the ratio of indentation loading slope at two different depths to determine the strain-hardening exponent and material-dependent constant [42].

The indentation technique has also been used to evaluate surface residual stress. Tsui et al. and Bolchakov et al. [43,44] demonstrated the effect of residual stress on the indentation curve and contact morphology with experimental verification and finite element analysis. The indentation load–depth curve shifts depending on the sign and scale of the residual stress. The optically measured contact area and Vickers hardness are invariant with residual stress in the load-controlled test. Theoretical models for residual stress evaluation have been proposed by many researchers. Suresh and Giannakopoulos [45] first suggested a relation between the ratios of residual stress and hardness and of the projected area in the stress-free and stressed state. Wang et al. [46] proposed a method to evaluate residual stress using the area between the stressed and stress-free indentation curve. These models are applicable for a wide range of materials with equibiaxial residual stress states. Atar [47] developed Suresh’s model, substituting a geometrical factor for material properties such as elastic modulus, yield strength, and ultimate strength. Lee and Kwon [48] separated the surface residual stress tensor into hydrostatic and deviatoric stress states. This model has the advantage of being applicable to a non-equibiaxial stress state without previous information on the mechanical properties of the material.

In this paper, HY-80 and HY-100 steels were T-joint welded by submerged-arc welding and repair-welded by flux-cored arc welding. The microstructures of the base metals and the heat-affected zone were investigated by an optical microscope and a hardness test. The yield strength and the residual stress of the welded coupons were evaluated using the macro-instrumented indentation test (MIIT). Uniaxial tensile testing was carried out for the base metals. The welding residual stress around the T-joint weld was compared with the results of X-ray diffraction, a representative non-destructive residual stress measurement technique. The change in yield strength with distance from the fusion line was evaluated. The accuracy and applicability of the instrumented indentation method for evaluating local yield strength and stress state were verified by the comparison with conventional methods. The effect of base metal (HY-80 and HY-100) and repair welds on yield strength and residual stress distribution are discussed. 

## 2. Theoretical Background

### 2.1. Evaluation of Tensile Properties Using Indentation

The relation between yield strength and Brinell hardness was proposed by Meyer as:

(1) $$\frac{L}{d^{2}}=C\cdot {\left(\frac{d}{D}\right)}^{m-2}$$

where *L* is the indentation load, *D* the indenter diameter and *d* the residual diameter, *C* the material constant, and *m* the Meyer index. George [49] proposed an empirical linear relationship between $\sigma_{y}$, the yield strength and *C*, the material constant:

(2) $$\sigma_{y}=\beta \cdot C$$

in which β depends on material class; β ranges from 0.3 to 15, and the corresponding *C* ranges from 800 to 3500 MPa for metallic materials. Kang et al. [42] derived the following equation using the stress field and constitutive equation to interpret the physical meaning of β:

(3) $$\sigma_{y}=\beta^{\prime }\cdot C^{\frac{1}{1-n}}$$

where $\beta^{\prime }$ is defined as a modified multiplying constant and depends on various mechanical parameters, such as elastic modulus, plastic constraint factor, and the strain-hardening exponent. In addition, it has been suggested that β′ has a strong correlation with the strain-hardening exponent among mechanical parameters that can be expressed as:

(4) $$\beta^{\prime }=f_{\beta^{\prime }}\left(p\right)=-13.43\cdot n^{3}+11.53\cdot n^{2}-3.42\cdot n+0.36$$

In addition, the relation between the strain-hardening exponent and the ratio of the loading slope is suggested to be:

(5) $$n=f_{n}\left(p\right)=-52.24\cdot p^{3}+228.18\cdot p^{2}-329.32\cdot p+157.31$$

where *p* is the ratio of the loading slope at two fixed contact radii (150 and 200 μm). Kang et al. [42] verified the modified Equations (3)–(5) for 13 metallic materials and found good agreement with the tensile testing results. Therefore, if we use the modified Meyer relation, the yield strength can be estimated by using only the indentation of the load–depth curve.

### 2.2. Evaluation of Superficial Residual Stress Using Indentation

**Figure 1:**
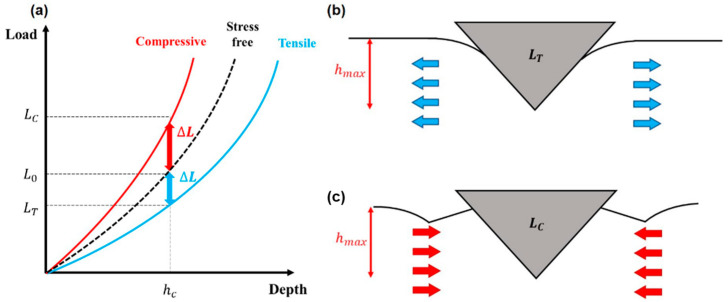
Effect of residual stress on (**a**) indentation curve; schematic diagram of contact morphology in (**b**) tensile stress state; (**c**) compressive stress state.

Figure 1 shows the effect of residual stress on the indentation load–depth curve [48]. The indentation curve shifts according to the sign and magnitude of the residual stress. With tensile residual stress, less load is required for the indenter to penetrate to a given indentation depth than in the stress-free state. Therefore, the indentation curve obtained in a tensile residual stress state is shifted downward in the indentation load–depth graph. Similarly, a compressive state curve is shifted in the opposite direction: a greater indentation load is needed than in the stress-free state for the same depth.

The effect of residual stress on the indentation curve is related to the change in the indentation surface morphology in a stress state [48]. The contact point between the indenter and the material is determined by the sink-in and pile-up behavior of the material around the indenter. These behaviors are influenced by the material’s stress state. As the stress state increases or decreases the contact height, the real contact area changes the load needed for the indenter to penetrate.

Lee and Kwon [48] quantified the relation between residual stress and load difference. They divided the surface residual stress components into hydrostatic and deviatoric stress, where $\sigma_{x}$, $\sigma_{y}$ are the two perpendicular residual stress components that are parallel to the surface. The z component of deviatoric stress, parallel to the indentation direction, was verified in various materials [20,21,22,24,38] to show an empirical relation with the load difference. As shown in Equation (7), the load difference divided by the optically measured contact area can be related to the surface residual stress components, when the ratio of the *x* and *y* stress components is *p*.

(6) $$\left(\begin{array}{ccc} \sigma_{x} & 0 & 0\\ 0 & \sigma_{y} & 0\\ 0 & 0 & 0 \end{array}\right)=\left(\begin{array}{ccc} \frac{1}{3}\left(\sigma_{x}+\sigma_{y}\right) & 0 & 0\\ 0 & \frac{1}{3}\left(\sigma_{x}+\sigma_{y}\right) & 0\\ 0 & 0 & \frac{1}{3}\left(\sigma_{x}+\sigma_{y}\right) \end{array}\right)+\left(\begin{array}{ccc} \frac{1}{3}\left(2\sigma_{x}-\sigma_{y}\right) & 0 & 0\\ 0 & \frac{1}{3}\left(-\sigma_{x}+2\sigma_{y}\right) & 0\\ 0 & 0 & -\frac{1}{3}\left(\sigma_{x}+\sigma_{y}\right) \end{array}\right)$$

(7) $$\sigma_{x}=\frac{3}{\left(1+p\right)}\cdot \frac{\Delta L}{A_{c}}\;(\mathrm{when}\;p=\frac{\sigma_{y}}{\sigma_{x}})$$

## 3. Materials and Welding Process

**Figure 2:**
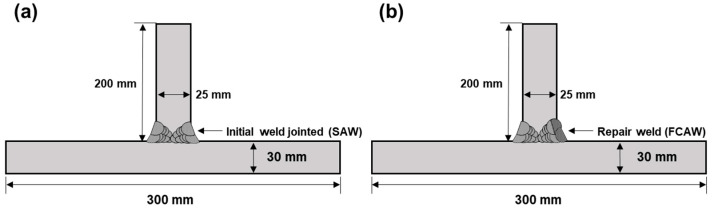
Schematic diagram of welding coupons (**a**) HY-80 and HY-100; (**b**) HY-100 repair weld.

Table 1 shows the chemical composition of the base metals. Two welded coupons (HY80 and HY100) were constructed with a 30 mm plate jointed on a 25 mm plate (Figure 2a). An additional HY100 steel coupon, HY100RP, was welded to investigate the effect of repair welding beside an initial weld (Figure 2b). Welding procedures followed the Welding Procedure Specification (WPS) provided by Daewoo Shipbuilding & Marine Engineering Co., Ltd., and welding conditions are specified in Table 2. Submerged-arc welding with AWS A5.23 EG (EFG mod.) wire (ϕ=4 mm) and EN ISO 14174:SA FB 1 55 AC H5 flux [50], and flux-cored arc welding with AWS A5.28 E80C-G H4 (ϕ=1.2 mm), were used for the initial and repair welds, respectively. A mixture of argon and carbon dioxide with the flow rate set at 25 L/min was used as the shielding gas. Both sides of the T-joint were welded in seven passes for the initial weld. For the repair weld, a section of the weld region on one side was removed by air carbon arc gouging using a carbon electrode (ϕ=10 mm), and an additional two passes were flux-cored arc welded. All welding processes were performed in the PA position.

## 4. Experiments

### 4.1. Microstructure Analysis and Hardness Testing

Samples for microstructure analysis were machined from the T-jointed weld coupon covering the fusion line and heat-affected zone. The prepared samples were mechanically polished up to 1 μm diamond suspension and chemically etched in a solution of 2% Nital, mixing 5 mL nitric acid with 95 mL ethanol. The area from the base region to the fusion line was observed by an optical microscope and a scanning electron microscope. The commercial microindentation system Micro-AIS (Frontics Inc., Seoul, Korea) provided the cross-sectional hardness distribution.

### 4.2. Macro-Instrumented Indentation Test (MIIT)

Test locations on the surface were selected through a line vertical to the welding direction of width 50 mm. The top and bottom coupon surfaces were mechanically polished with sandpaper and alumina colloidal silica suspension to minimize the effect of surface roughness on indentation test data.

**Figure 3:**
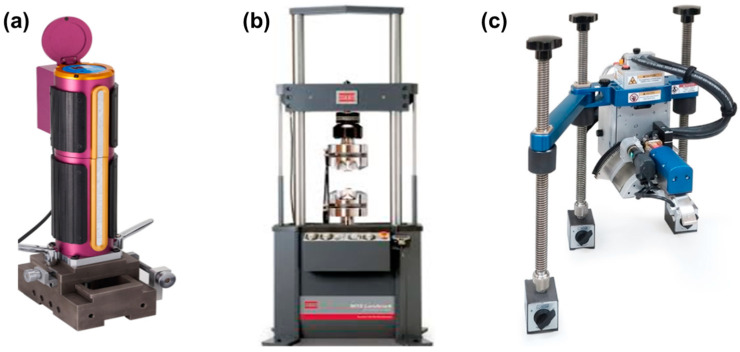
Instruments used: (**a**) AIS 3000-HD; (**b**) Servohydraulic Test Systems; (**c**) Xstress 3000 G2R.

The AIS 3000-HD system (Frontics Inc., Seoul, Korea; Figure 3a) with a load and depth resolution 2 gf and 0.1 μm was used for the MIIT. The test machine was well verified and calibrated following ISO 14577–2 [51]. Prior to the indentation test on the coupons, at least five pretests were performed on a reference block to confirm the repeatability of the test data.

A tungsten carbide spherical indenter with a radius of 250 μm was used for tensile property measurement. The test was controlled with a 150 μm maximum depth. Multiple loading and 50% unloading at every 10 μm depth was performed. Residual stress measurement was performed using a Vickers indenter with 50 kgf controlled single loading and unloading; loading and unloading rates were 0.3 mm/min for all indentation tests, and at least three repeatable test data points for each test location were obtained at a minimum interval of 3 mm.

Stress-free state curves were obtained from the area in which the residual stress was relieved by electrical discharge machining. Cutting was done carefully to minimize the change in mechanical properties or microstructure at a speed of 10 mm/h with a wire thickness of 100 μm.

### 4.3. Uniaxial Tensile Testing and X-ray Diffraction

Tensile specimens were machined from each coupon following ASTM E8/E8M [52]. The uniaxial test was performed with a 100 kN capacity MTS Landmark^®^ Servohydraulic Test System (MTS System Corp., Eden Prairie, MN, USA), shown in Figure 3b, with a constant crosshead speed of 2 mm/min at 25 °C.

Residual stress measurement using X-ray diffraction was performed for reference prior to indentation. The mechanically polished surface was electrically polished with a 94% acetic acid and 6% perchloric acid solution. X-ray residual stress measurement was performed using the Xstress 3000 G2R system (Stresstech, Ltd., Jyväskylä, Finland; Figure 3c with the parameters summarized in Table 3). The test locations were exactly the same as those in the indentation test for residual stress measurement. 

## 5. Results and Discussion

### 5.1. Microstructural Analysis and Hardness Test

**Figure 4:**
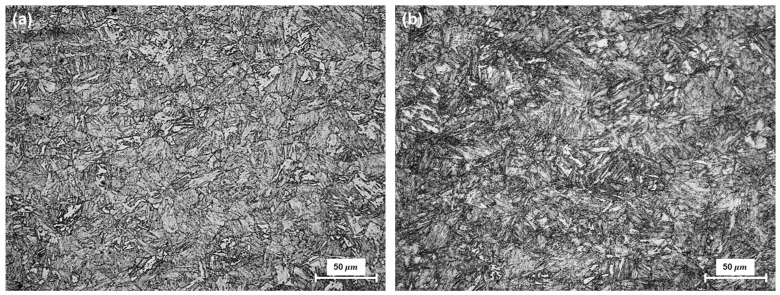
Microstructure of base metals: (**a**) HY-80; (**b**) HY-100 steels.

Mixtures of granular bainite and lath-like tempered martensite with irregular carbide precipitation, generally observed in low-carbon steel, were seen in the base metal of the HY80 and HY100 coupons (Figure 4) [2,3]. These microstructures are formed by quenching followed by tempering. HY100 contains a larger area fraction of tempered martensite than HY80, as is consistent with the hardness results. The average hardness of the HY-100 base metal (248 HV) was approximately 10% greater than that of the HY-80 base metal (232 HV).

**Figure 5:**
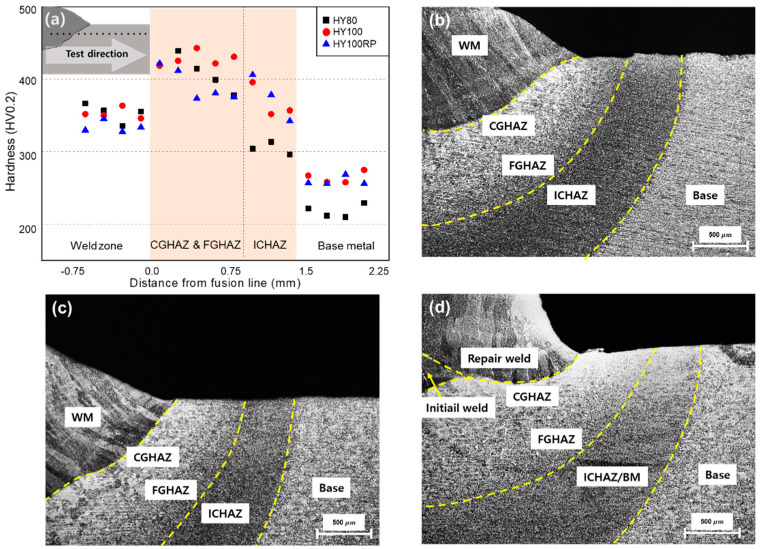
(**a**) Hardness distribution around the fusion line; overall microstructure of (**b**) HY80; (**c**) HY100; (**d**) HY100RP.

The hardness was measured 200 μm from the top surface on the cross-section, as shown in Figure 5a, which is consistent with the location of the microstructural analysis. The locations were determined to check the microstructure close to the top surface, where the yield strength and residual stress were evaluated as in Section 5.2 and Section 5.3. The heat-affected zone (HAZ) was observed from the fusion line to the base metal and divided into three subregions to analyze microstructures with distance from the fusion line (Figure 5) [3,10,53]. The coarse-grained heat-affected zone (CGHAZ)—the area adjacent to the fusion line—shows a coarse grain size transformed over the austenitizing temperature [6,54]. The fine-grained heat-affected zone (FGHAZ) shows a smaller grain size than CGHAZ because of the lower heat input. The intercritical heat-affected zone (ICHAZ), where the maximum temperature is below the austenitizing temperature, is the partially transformed region.

**Figure 6:**
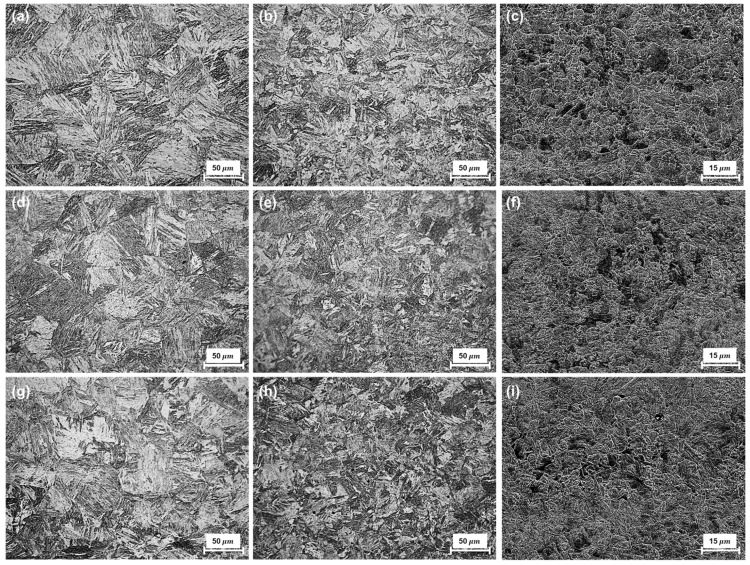
CGHAZ, FGHAZ, and ICHAZ microstructures of (**a**–**c**) HY80; (**d**–**f**) HY100; (**g**–**i**) HY100RP.

Untempered lath martensite and granular bainite microstructures were observed in CGHAZ of all three coupons, with coarse prior-austenite grains contributing to the increase in hardness. The microstructures of FGHAZ show similar morphology, a mixture of martensite and bainite, with a finer grain size. The difference in heat input between the two regions changed the maximum temperature and prior-austenite grain size. The prior-austenite grain size increased the volume fraction of martensite by lowering the martensitic transformation temperature. The maximum hardness was found in these subregions of the HAZ, and the average hardness of HY80 and HY100 was approximately 420 HV. ICHAZ, which occupies almost half the HAZ, was clearly different from the other HAZ. Acicular ferrite and polygonal ferrite reduced the hardness to less than 400 HV (Figure 6) [10]. No cracks were found in the three coupons as a result of welding.

### 5.2. Tensile Properties Using Indentation

The yield strengths of HY80 and HY100 were evaluated using the indentation technique, following a process based on Section 2.1. Load–depth curves can be converted into contact radius–load curves using the geometric formula for a spherical shape. The strain- hardening exponent *n* is calculated by Equation (5) with the ratio between the slopes at contact radii of 150 and 200 μm. Using *n*, $\beta^{\prime }$ (the modified multiplying constant) is obtained following Equation (4). The material constant *C* is calculated by substituting the indentation parameters and indenter information into Equation (1). The sample calculation process is summarized in Table 4.

**Figure 7:**
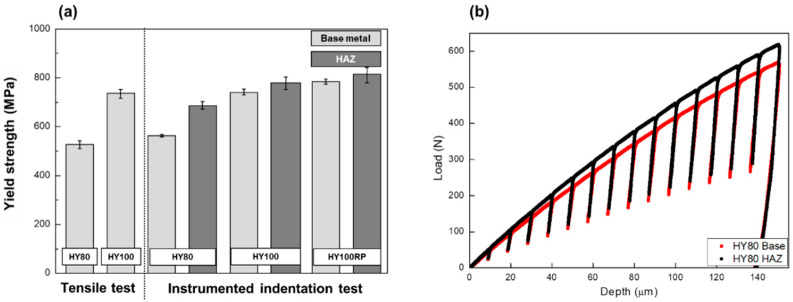
(**a**) Yield strength results in the uniaxial tensile test and the MIIT; (**b**) indentation load–depth curves for the base region and HAZ in HY80 coupon.

Uniaxial tensile testing provided the yield strength of the base metals for verification. The average yield strength from the test of HY-80 and HY-100 steel was 579 MPa and 735 MPa, both 5% higher than the minimum requirements. The average yield strengths in the indentation test are comparable to the tension test data. The base regions of HY80 and HY100 show 562 MPa and 738 MPa MIIT yield strength, which are within the range of variation. HY100RP shows an approximately 5% higher MIIT yield strength than in the uniaxial tensile test. The standard deviations of the MIIT yield strength were less than 35 MPa at comparable levels to the uniaxial tension test (Figure 7).

The yield strength of the HAZ (Figure 7) was evaluated by the indentation test, taking advantage of the indentation’s local measurement capabilities. The strength was increased by approximately 40 MPa in the HAZ over the base metal. The change in local microstructure affected the indentation load–depth curve. Figure 7b shows the difference between the indentation load–depth curves of the base region and the HAZ in the HY80 coupon. Higher load is required for the HAZ than for the base with increasing depth, so that the loading slope increased: approximately 5% higher $\beta^{\prime }$ was calculated for the HAZ than for the base. 

### 5.3. Superficial Residual Stress Using Indentation

**Figure 8:**
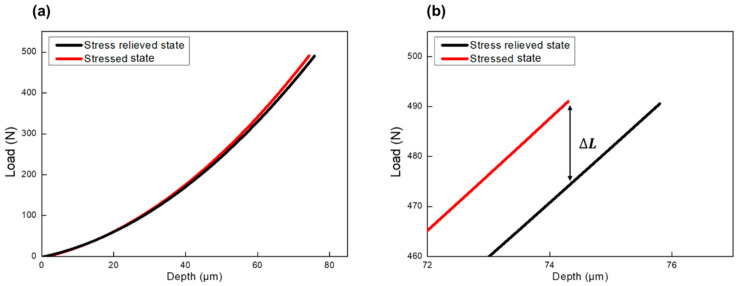
Indentation loading curves of HY100 for (**a**) stress-relieved state (black), stressed state (red) (**b**) enlarged curves at the maximum indentation load.

The residual stress in the region from the fusion line to the base was evaluated using the indentation technique, following a process based on Section 2.2. After the indentation tests for a stressed state, an adjacent location was tested again for the stress-free state through unidirectional stress relief. The hardness difference between the stressed and stress-free points should not exceed 3% to minimize mechanical property change, which could alter the stress state evaluation. The load difference between the stressed and stress-free state was measured at the maximum indentation depth. Finally, the stress state was calculated using the measured load difference and contact area using Equation (7). The stress ratio, *p*, is zero because the unidirectional relieved stress is evaluated. The sample calculation process is summarized in Table 5. Figure 8 shows the indentation curves of HY100 for the stress-relieved state and the stressed state as an example.

**Figure 9:**
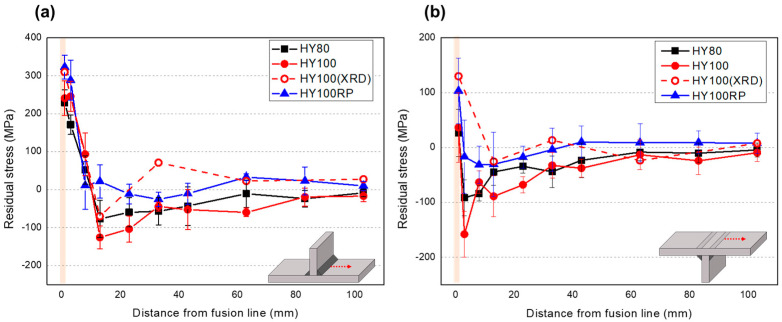
Residual stress distribution using the MIIT and X-ray diffraction method of three coupons on (**a**) top surface (**b**) bottom surface.

The side of the T-joint on which a seven-pass welding was done before the other T-joint side was measured for all three coupons. The asymmetry of residual stress distribution was not considered. The top and bottom surfaces of the coupons were measured at the symmetrical position. The MIIT residual stress data points in Figure 9 are the averaged results of more than five test points at each location. X-ray diffraction was used for five locations on the HY100 coupon to verify the MIIT residual stress evaluation method, as shown in Figure 9.

The results for the top surface show a similar trend between the two residual stress measuring methods. The maximum tensile residual stress was detected in the HAZ, and the stress shows saturating distribution as the distance from the fusion line toward the base region increases. The maximum difference between the two methods was 115 MPa 33 mm away from the fusion line. The bottom surface residual stresses were also distributed with similar peaks and saturation trend, but the maximum tensile stresses measured by X-ray and MIIT were reduced to 130 and 47 MPa, respectively. The minimum residual stress value, 158 MPa compressive stress, was found close to the HAZ. The difference in the results of the different methods can be explained by the sensing depth and area. The X-ray method detects a 1 mm diameter circle with a penetration depth of up to approximately 30 μm. The indentation load–depth curve, on the other hand, is affected by the stress at depths up to ten times the maximum indentation depth.

The stress distribution by the indentation testing method has a similar trend on all three coupons, as shown in Figure 9. HY100RP shows the highest tensile residual stress (323 and 103 MPa on the top and on the bottom surface), which could be considered an effect of the repair weld. 

## 6. Conclusions

This paper investigated the mechanical properties and residual stress in a T-joint weld fabricated from HY-80 and HY-100 and a T-joint weld of HY-100 repaired with flux-cored arc welding. The effects of the base metal and repair welding were analyzed by comparing the evaluation results and by microstructural analysis. The results are summarized below:

- The heat-affected zone of the three coupons showed similar microstructural characteristics with distance from the fusion line. The coarse-grained HAZ and finer-grained HAZ mainly contained untempered martensite and granular bainite, which increased the hardness. The intercritical HAZ contained a mixture of acicular ferrite and polygonal ferrite with a hardness of under 400 HV.
- MIIT can provide local characterizations of mechanical properties and residual stress distributions. The distribution of the yield strength on the top surface of the T-jointed coupons was evaluated. Indentation measurements provided yield strength results comparable with the tensile test results for the base metal, where it was not significantly affected by welding. The yield strength of the HAZ was evaluated, taking advantage of the local measurement characteristics of the indentation technique. The HAZ showed an increase in properties ranging from 3% to 10%. The shift in the indentation load–depth curve explains the change in the final output.
- MIIT measurement provides a quantitative evaluation of the welding residual stress distribution. The X-ray diffraction method was applied to the HY100 coupon to verify the MIIT residual stress evaluation method. The two methods yielded stress distributions in the HAZ to 100 mm, with similar trends within the 150 MPa range. The difference between the X-ray diffraction method and the indentation method can be explained by the differences in the stress sensing area and depth. Further study to clarify the measuring depth and area of the indentation residual stress method is needed for accurate comparison of the stress results with other techniques.
- All three of the coupons showed similar residual stress distribution trends. HY100RP showed the highest tensile residual stress adjacent to the fusion line, which was an effect of the repair weld.

## Figures and Tables

**Table 1:** Chemical composition of base metals.

Material	C	Si	Mn	P	S	Cr	Ni	Cu	Mo	Ti	V
HY-80	0.14	0.27	0.26	0.009	0.002	1.57	3.069	0.021	0.522	0.0022	0.004
HY-100	0.16	0.26	0.24	0.008	0.001	1.503	3.012	0.023	0.399	0.0019	0.018

**Table 2:** Welding parameters.

Parameters	SAW	FCAW
Welding current (A)	500	180
Arc voltage (V)	30	23
Welding speed (mm/s)	10	3.3
Heat input (kJ/mm)	1.5	1.24

**Table 3:** Parameters for X-ray measurement using Xstress 3000 G2R.

Radiation Source	CrKα
Wavelength (Å)	λ = 2.29106867
Power	30 kV and 6.7 mA = 210 W
2θ range	125–1620
Miller indices and Bragg’s angle	{211} set of planes; Bragg’s angle = 156˚
Aperture size	1 mm diameter
Analysis	$sin^{2}\Psi$ method

**Table 4:** Example of calculation process evaluating yield strength.

Material	*p*	*n*	$C^{\frac{1}{1-n}}$	$\beta^{\prime }$	Yield Strength (MPa)
HY80	1.3875	0.120	2120	0.092	556.9
HY100	1.3775	0.099	2566	0.1214	737.6

**Table 5:** Example of the calculation process evaluating stress state.

Parameter	Point 1	Point 2	Point 3
Load difference, ΔL (N)	−16.2	−10.9	−17.4
Contact area, $A_{c}$ (${\mathrm{mm}}^{2}$)	0.353	0.359	0.355
Residual stress, σ (MPa)	−138	−91	−147

## Data Availability

The data presented in this study are available on request from the corresponding author.
